# Lesion Localization and Prognosis Using Electrodiagnostic Studies in Facial Diplegia: A Rare Variant of Guillain-Barre Syndrome

**DOI:** 10.7759/cureus.25047

**Published:** 2022-05-16

**Authors:** Liaquat Ali, Mohammed Alhatou, Gholam Adeli, Osama Elalamy, Yasin Zada, Imran Mohammed, Muhammad Sharif, Memon Noor Illahi, Muhammad Naeem, Ambreen Iqrar

**Affiliations:** 1 Neurology, Hamad General Hospital, Doha, QAT; 2 Neurology, Weill Cornell Medicine-Qatar, Doha, QAT; 3 Internal Medicine, Hamad General Hospital, Doha, QAT; 4 Neurology, Aga Khan Health Service, Karachi, PAK

**Keywords:** amplitude degeneration index (adi), guillain-barre syndrome (gbs), nerve conduction study (ncs), facial diplegia (fd), acute motor axonal neuropathy (aman)

## Abstract

Background

The etiology of facial nerve palsy is diverse and includes herpes zoster virus, Guillain-Barre syndrome (GBS), otitis media, Lyme disease, sarcoidosis, human immunodeficiency virus, etc. The lower motor neuron type facial nerve palsy is usually caused by an ipsilateral facial nerve lesion; however, it may be caused by a central lesion of the facial nerve nucleus and tract in the pons. Facial diplegia is an extremely rare condition that occurs in approximately 0.3% to 2.0% of all facial palsies. Electrodiagnostic studies including direct facial nerve conduction, facial electromyography (EMG), and blink reflex studies are useful for the prognosis and lesion localization in facial nerve palsy.

Methodology

This retrospective, observational study was conducted at the Neurophysiology Unit, Hamad General Hospital, Doha, Qatar. This study included 11 patients with bilateral facial weakness who visited for electrodiagnostic studies in the neurophysiology laboratory.

Results

In total, eight (72.7%) patients had facial diplegia, eight (72.7%) had hypo/areflexia, seven (63.6%) had facial numbness, and five (45.5%) had cerebrospinal fluid albuminocytological dissociation. The most frequent cause of facial diplegia in this study was GBS (81.9%). Direct facial nerve conduction stimulation showed that nine (81.8%) patients had bilateral facial nerve low compound muscle action potential amplitudes. The bilateral blink reflex study showed that eight (88.8%) patients had absent bilateral evoked responses. Finally, the EMG study showed that five (55.5%) patients had active denervation in bilateral sample facial muscles.

Conclusions

Bilateral facial nerve palsy is an extremely rare condition with a varied etiology. Electrodiagnostic studies are useful in detecting the underlying pathophysiologic processes, prognosis, and central or peripheral lesion localization in patients with facial diplegia.

## Introduction

In the early 1800s, Sir Charles Bell first reported facial nerve palsy [1]. Sir Bell first described facial paralysis caused by trauma to the peripheral branches of the facial nerve. Bell’s palsy is defined as an acute peripheral facial nerve palsy of unknown cause and accounts for approximately half of all cases of facial nerve palsy [2,3]. The annual incidence ranges between 13 and 34 cases per 100,000 individuals [4]. The risk of Bell’s palsy is three times greater during pregnancy, especially in the third trimester or in the first postpartum week; however, there is no race, geographic, or gender predilection [5]. Diabetes is present in 5% to 10% of facial nerve palsy cases [6]. Facial nerve palsy is caused by diverse disorders such as herpes zoster infection, Guillain-Barre syndrome (GBS), otitis media, Lyme disease, human immunodeficiency virus (HIV), etc. Peripheral facial palsy is a clinical syndrome. Herpes simplex virus activation is the most likely cause of Bell’s palsy, and herpes zoster is the second most common viral infection associated with facial nerve palsy [7]. Recurrent attacks of idiopathic facial palsy on either the ipsilateral or contralateral side have been observed in 7-15% of patients [8].

Peripheral lesion of the facial nerve that causes partial or complete hemifacial paralysis of one side of the face including the forehead is usually caused by ipsilateral facial nerve lesions; however, other possible causes include an ipsilateral central lesion of the facial nerve nucleus or intra-axial tract in the pons (brainstem). The facial nerve and facial muscles are the final common pathway in central activation for both voluntary and emotional activation. Therefore, dissociation of voluntary movement of facial muscles to command from spontaneous emotional facial expressions, such as in spontaneous smiling, indicates an upper motor neuron facial lesion, whereas the absence of this dissociation indicates a lower motor neuron facial lesion [9].

Facial diplegia (bilateral facial paralysis) is an extremely rare condition that occurs with various central or peripheral diseases such as sarcoidosis, Lyme disease, GBS, Melkersson-Rosenthal syndrome, tuberculous meningitis, leptomeningeal lymphomatosis/carcinomatosis, some neuromuscular junction disorders, and in various muscular dystrophies [10]. It occurs in approximately 0.3% to 2.0% of facial palsy cases [11]. The electrodiagnostic evaluation of the facial nerve may involve the direct facial nerve conduction study, blink reflex study, and needle electromyography (EMG) study. Facial nerve direct nerve conduction stimulation studies exclusively assess the distal segment of the nerve, whereas blink reflex studies evaluate the proximal segment of the facial nerve, in addition to the whole blink reflex arc between the trigeminal nerve, brainstem, and facial nerves [12-14]. In general, facial nerve compound muscle action potential (CMAP) amplitude of 50% to 75% lower than the contralateral normal side is associated with a poor prognosis, long recovery time, and a high chance of aberrant reinnervation [15]. A small concentric EMG needle should always be used to study facial and trigeminal nerves innervated muscles to examine the dysfunction of the cranial nerve. Neuroimaging such as magnetic resonance imaging (MRI) of the brain and cranial nerves is justified if atypical physical signs are noted, there is a slow progression over three weeks or no improvement at four months, and in the presence of a facial twitch or spasm preceding facial weakness which may be due to neoplastic nerve infiltration or irritation [16].

## Materials and methods

This observational, retrospective study was conducted in the Neurophysiology Department of Hamad General Hospital, Qatar, after review and approval by the institutional review board (IRB# 1-19-239). The study included 11 patients with clinical bilateral facial paralysis/facial diplegia who visited the Neurophysiology Unit for electrodiagnostic investigation from January 1, 2017, to May 14, 2019. Patients with a history of recurrent Bell’s palsy, Ramsay Hunt syndrome, traumatic facial nerve palsy, upper motor neuron type facial nerve palsy, and presence of an intracardiac pacemaker or defibrillator were excluded. Patients with bilateral facial nerve palsy or facial diplegia, either symmetrical or asymmetrical, were included. Clinically, facial nerve palsy can be classified using the House-Brackmann scale from grade 1 to 6. These stages correspond with the pathologic findings of neurapraxia (grade 1), axonotmesis (grades 2-3), neurotmesis (grade 4), and partial and complete transection of the facial nerve (grades 5-6) [17]. The neurophysiological studies were performed within two weeks of the onset of facial diplegia using the key point V5.03 four-channel amplifier (Medtronic, Minneapolis, MN, USA). Direct facial nerve conduction studies were performed with percutaneous stimulation of the facial nerve and recording from the facial muscles (nasalis or orbicularis oculi) bilaterally. The supramaximal facial nerve stimulation of amplitude was recorded from facial nerve innervated muscles to measure CMAP amplitude of the facial nerve; amplitude degeneration index (ADI) was calculated using the following equation: 100-(CMAP amplitude affected side/unaffected side × 100). Blink reflex studies were performed with stimulation of the supraorbital nerve of the trigeminal nerve, recorded from orbicularis oculi muscles bilaterally with the patient lying down in a quiet room. The evoked muscle action potential (Ipsilateral R1 and R2, and contralateral R2 response) responses were defined as normal, delayed (ipsilateral R1 latency >13 ms and R2 latency >41 ms and contralateral R2 latency >44 ms), and absent [18]. Concentric EMG needles were examined of facial nerve innervated muscles such as frontalis, orbicularis oculi, orbicularis oris, mentalis, and trigeminal nerve innervated muscle such as masseter on the affected side. The EMG findings included a degree of insertional activity, spontaneous activity, and voluntary activity, and recruitment and interference patterns were noted. Wakerley diagnostic criteria for GBS with facial diplegia were used, and Albers diagnostic criteria for acquired inflammatory demyelinating polyneuropathy (AIDP) of GBS were applied [19,20].

The primary aim of this study was to assess axonal loss with the help of facial nerve CMAP amplitude difference on side to side compared to normative data in direct facial nerve conduction study, blink reflex study, and EMG study to determine the prognostic markers for facial diplegia, evaluate diverse causes, and to localize lesions. Descriptive statistics were used to summarize and determine the sample characteristics and distribution of various considered parameters including demographic factors, diagnostic factors, clinical features, follow-up outcomes, and other related features of the patients. The normally distributed data and results were reported as mean and standard deviation (SD) with the corresponding 95% confidence interval (CI). The remaining results were reported as the median and interquartile range (IQR). Categorical data were summarized using frequencies and percentages. Pictorial presentations of the key results were made using appropriate statistical graphs. A two-sided P-value of <0.05 was statistically significant. All statistical analyses were done using SPSS version 24.0 (IBM Corp., Armonk, NY, USA) and Epi Info 2000 (Centers for Disease Control and Prevention, Atlanta, GA, USA).

## Results

In total, 11 patients with facial diplegia who visited the Neurophysiology Department of Hamad General Hospital for electrodiagnostic studies underwent nerve conduction, blink reflex, and EMG studies. Patients’ demographic data are shown in Table 1, and clinical characteristics are shown in Table 2.

**Table 1:** Demographic data of the patients. The mean age, gender, and nationality of the patients.

Demographic variables	Frequency (n)	Percentage (%)
Age (years)	Mean = 36 (range = 21–68 years)	
Male	7	63.6%
Female	4	36.4%
Indian	3	27.27%
Bangladesh	1	9.09%
Filipino	2	18.18%
Kuwaiti	1	9.09%
Pakistani	1	9.09%
Sudani	1	9.09%
Nigerian	1	9.09%
Syrian	1	9.09%

**Table 2:** Clinical manifestations. Neurologic symptoms and signs included facial diplegia, hypo/areflexia, facial numbness, dysarthria, ataxia, dysphagia, limb numbness, ophthalmoplegia, upper extremity weakness, and lower extremity weakness.

Symptoms/Signs	Frequency (n)	Percentage (%)
Facial diplegia	8	72.7%
Hypo/Areflexia	8	72.7%
Facial numbness	7	63.6%
Dysarthria	6	54.5%
Limb numbness	4	36.4%
Dysphagia	4	36.4%
Ataxia	4	36.4%
Ophthalmoplegia	3	27.35
Upper extremity weakness	2	18.2%
Lower extremity weakness	1	9.1%

The mean age of the patients was 36 (range = 21-68 years), and 63% (seven) of the patients were men and 37% (four) were female. Neurologic symptoms and signs included 72.7% (eight) facial diplegia, 72.7% (eight) hypo/areflexia, 63.6% (seven) facial numbness, 54.5% (six) dysarthria, 36.4% (four) ataxia, dysphagia and limb numbness each, 27.3% (three) ophthalmoplegia, 18.2% (two) upper extremity weakness, and 9.1% (one) lower extremity weakness (Table 2). Table 3 presents the findings of the lumbar puncture analysis; 45.5% (five) of patients had high cerebrospinal fluid (CSF) protein levels, 45.5% (five) had albuminocytological dissociation, 18.2% (two) had pleocytosis with <50 neutrophil counts, and 9.1% (one) of patients were anti-GQ1b positive. As shown in Table 4, 81.9% (nine) of patients had GBS, 9.1% (one) had lymphomatosis carcinomatosis, 9.1% (one) had trigeminal motor neuropathy, and six patients had the GBS variant of facial diplegia, one case each of pharyngeal cervical brachial, acute motor axonal neuropathy (AMAN) and Miller-Fisher syndrome.

**Table 3:** Lumbar puncture findings. Lumbar puncture analysis showed high CSF protein levels and albuminocytological dissociation. LP: lumbar puncture; CSF: cerebrospinal fluid

LP (results of 10 patients)	Frequency (n = 10)	Percentage (%)
High protein (range = 0.59–1.46)	5	45.5%
Albuminocytological dissociation	5	45.5%
Pleocytosis (range = 22 and 28 neutrophils)	7	18.2%
AntiGQ1b+	1	9.1%
Low CSF sugar	0	0
Oligoclonal bands+	0	0

**Table 4:** Diagnosis and treatment. GBS: Guillain-Barre syndrome; AMAN: acute motor axonal neuropathy; IVIG: intravenous immunoglobulin

Underlying diagnosis and treatment	Frequency (n)	Percentage (%)
GBS	9	81.9%
Lymphomatosis carcinomatosis	1	9.1
Trigeminal motor neuropathy	1	9.1
GBS variants		
Facial diplegia	6	6
Pharyngeal-cervical-brachial	1	1
Miller Fisher syndrome	1	1
AMAN	1	1
Treatment administered		
IVIG	8	72.7%
Chemotherapy	1	2.4

Facial, upper, and lower extremity nerve conduction study and blink reflex electrodiagnostic study showed that, in 81.8% (nine) of patients, the direct facial nerve conduction study was abnormal. In 88.8% (eight) of patients, the blink reflex study showed bilateral absent evoked responses. In 30% (three) of patients, the nerve conduction study of the upper limbs was abnormal, and in 20% (2) nerve conduction study of the lower limbs was abnormal (Table 5). Repetitive nerve stimulation (RNS) of four patients did not show a significant decrement response (>10%) (not shown in Table 5). The EMG study showed that 55.5% (five) of needle EMG examinations were abnormal with active denervation (fibrillation and positive sharp waves) in bilateral sampled facial muscles, while one patient showed chronic neurogenic unit on the left-sided masseter muscles of mastication (Table 6).

**Table 5:** Nerve conduction study of facial, upper, and lower extremity nerves and blink reflex study findings and diagnosis. Overall, 81.8% (9) of direct facial nerve conduction and bilateral blink reflex were abnormal. (Normal facial nerve CMAP amplitude from orbicularis oculi = >1 mV, normal distal latency of facial nerve = <3.1 ms.) AMAN: acute motor axonal neuropathy; AIDP: acute inflammatory demyelination polyneuropathy; MFS: Miller Fisher syndrome; FD: facial diplegia

Facial nerve conduction study cases	Right amplitudes (N = >1 mV)	Left amplitudes (N = >1 mV)	Right distal latencies (N = <3.1 ms)	Left distal latencies (N = <3.1 ms)	Upper and lower extremity nerve conduction study	Blink reflex study	Diagnosis
1	2	1.3	3.6	3.6	Normal	Absent bilateral	Facial diplegia-GBS
2	1.1	1.6	3.2	3.2	Normal	Absent bilateral	Facial diplegia-lymphomatosis carcinomatosis
3	0.5	0.7	3.4	3.5	Normal	Absent bilateral	Facial diplegia-GBS
4	0.5	0.3	3.4	3.5	Abnormal (demyelination)	Absent bilateral	Facial diplegia-GBS-(AIDP)
5	0.8	0.3	3.7	3.5	Abnormal (axonal)	Not performed	Facial diplegia-GBS-(AMAN)
6 (early study on day 2 of FD)	4.5	4.5	2.5	3.6	Normal	Absent bilateral	Facial diplegia-GBS
7	0.7	0.8	3.1	3.6	Abnormal (axonal spinal accessory nerve)	Not performed	Pharyngeal cervical brachial-GBS
8	2.4	2.0	3.7	3.4	Normal	Absent bilateral	Facial diplegia-GBS
9	Absent	0.3	Absent	3.8	Normal	Absent bilateral	Facial diplegia-GBS
10	0.3	0.7	3.2	3.9	Normal	Absent bilateral	MFS-GBS (AntiGQ1b+ve)
11	2.5	2.6	2.8	2.3	Not performed	Normal	Left axonal motor trigeminal neuropathy (diagnosed by EMG study of abnormal trigeminal muscle findings)

**Table 6:** Needle EMG of facial muscles. Needle EMG examinations showed abnormal findings with active denervation (fibrillation, PSW) in bilateral facial muscles (55.5%, 5) while one patient showed a chronic neurogenic unit in the left masseter muscles. EMG: electromyography; PSW: positive sharp wave; MUAP: motor unit action potential

Facial muscles EMG cases	Spontaneous activity	MUAP morphology	Recruitment	Interface patterns
1	+2 fibrillation, +2 PSW	Normal	Reduced with rapid fire rate bilateral	Reduced
2	+2 fibrillation, +2 PSW	No activation	No activation	No activation
3	+2 fibrillation, +2 PSW	No activation	No activation	No activation
4	No	Normal	Reduced with rapid fire rate bilateral	Reduced
5	+1 fibrillation, +2 PSW	Normal	Reduced with rapid fire rate bilateral	Reduced
6	No	Normal	Reduced with rapid fire rate bilateral	Reduced
7	+1 fibrillation, +1 PSW	No activation	No activation	No activation
8	No	Normal	Reduced with rapid fire rate bilateral	Reduced
9	+2 fibrillation, +2 PSW	Normal	Reduced with rapid fire rate bilateral	Reduced
10	+2 fibrillation, +2 PSW	Normal	Reduced with rapid fire rate bilateral	Reduced
11	No	Chronic neurogenic unit in the left masseter muscle	Reduced with rapid fire rate left masseter	Reduced

MRI of the head with gadolinium showed that three patients had bilateral facial nerve enhancement and one lymphoma patient showed bilateral facial nerve enhancement likely due to neoplastic infiltration. Moreover, one patient had atrophy of the left muscles of mastication and subtle prominence of the mandibular division of the left trigeminal nerve post-dental extraction (Table 7).

**Table 7:** MRI of the head and spine findings. MRI of the head and spine with gadolinium showed bilateral enhancement of the facial nerves in three patients and atrophy of the left muscles of mastication in one patient. MRI: magnetic resonance imaging; IAC: internal acoustic canal

	Findings
Case 2	Bilateral enhancement of the facial nerves in the IAC and right intra-canalicular segment of the facial nerve, with the possibility of lymphoma infiltrations
Case 6	Bilateral focal enhancement along the proximal inner canalicular regions of the facial nerves
Case 9	Bilateral facial nerve enhancement and contrast enhancement around conus medullaris and cauda equina
Case 11	Atrophy and fatty replacement of the left side muscles of mastication, as well as subtle prominence of the left foramen ovale with subtle prominence of the mandibular division of the left trigeminal nerve

**Figure 1:**
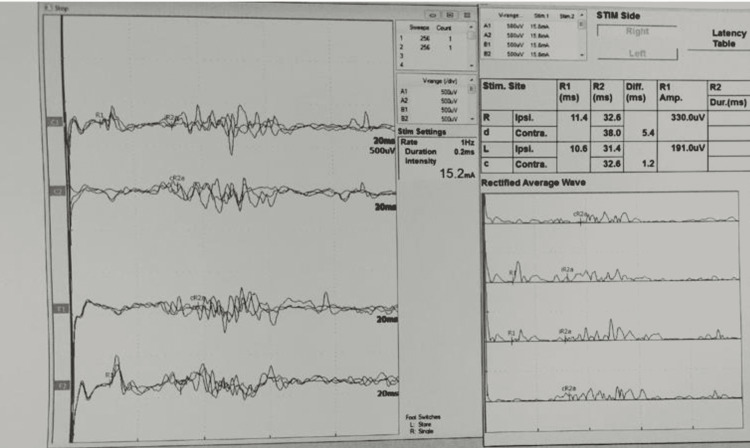
Blink reflex electrodiagnostic study. Normal bilateral blink reflex study showing normal minimal latencies of ipsilateral R1 and R2 and contralateral R2 (R = response).

**Figure 2:**
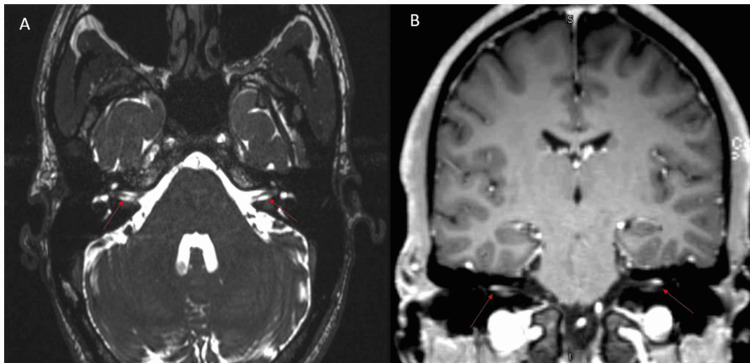
MRI brain with gadolinium. MRI brain axial T2-weighted image of the IAM demonstrating normal caliber facial nerves bilaterally (red arrows in A) and coronal T1 post-contrast image of the IAM demonstrating enhancement of distal intra-canalicular facial nerves (red arrows B). IAM: internal acoustic meatus; MRI: magnetic resonance imaging

Figure 1 shows a normal bilateral blink reflex electrodiagnostic study with normal minimal latencies of ipsilateral R1 and R2 and contralateral R2 recorded from both orbicularis oculi simultaneously. Figure 2 (of case 9) shows an axial T2-weighted image of the internal acoustic meatus (IAM) demonstrating normal caliber bilateral facial nerves (shown using red arrows in Figure 2A), coronal T1 post-contrast image demonstrates enhancement of the bilateral distal intra-canalicular facial nerves, as well as labyrinthine and proximal tympanic segments (shown using red arrows in Figure 2B). 

## Discussion

This study included 11 patients with facial diplegia, bilateral facial paralysis, or paresis with or without other neurological deficits. The most common neurological signs and symptoms were bilateral facial weakness, hypo or areflexia, facial numbness, bulbar symptoms (dysarthria, dysphagia), limb numbness, and ataxia. Out of the 11 facial diplegia patients, nine (81.9%) had GBS, including the different variants of GBS such as facial diplegia, Miller Fisher syndrome, pharyngeal-cervical-brachial, and AMAN. All patients underwent an MRI of the brain with contrast, of whom three patients showed bilateral facial nerve enhancement. The most common electrodiagnostic study findings included low CMAP amplitudes on direct facial nerve stimulation suggestive of the distal segment of nerve dysfunction, bilateral absent blink reflex suggestive of the proximal segment of the motor facial nerve (or sensory trigeminal nerve), and demyelinating pathophysiology and active denervation on needle EMG of facial muscles with normal muscles of mastication suggestive of facial nerve peripheral lesion (lower motor neuron). In facial diplegia patients, bilateral greater than 50-75% low facial nerve CMAP amplitudes on side-to-side comparison or greater than 50-75% ADI as well as moderate-to-severe active denervation in needle EMG were suggestive of acute severe axonal loss and were poor prognostic markers [21].

Dr. Allan H. Ropper from Massachusetts General Hospital, Boston, reported seven patients with four syndromes of the acute regional variant of GBS including (i) four patients with facial diplegia and distal limb paresthesia, (ii) one with cranial nerve sixth palsy and distal paresthesia, (iii) one with combined Miller Fisher syndrome and pharyngeal-cervical-brachial weakness, and (iv) one with bilateral lumbar polyradiculopathy. These acute regional variants of GBS may suggest that the pathologic process occurs in the same single or contiguous groups of bilateral facial or other cranial or spinal nerves in the peripheral nervous system [22].

In a literature review of facial diplegia as a variant of GBS, Susuki et al. reported 22 patients with acute progressive facial diplegia, distal limb paresthesia, hypo or areflexia, and absence of other cranial neuropathies, ataxia, or limb weakness, consistent with the facial diplegic variant of GBS. Overall, 18 (86%) patients had had infectious symptoms preceding the onset of GBS, and the most frequent (35%) positive serology test was anti-cytomegalovirus immunoglobulin M. All patients had CSF albuminocytologic dissociation, and 64% (14) of nerve conduction studies showed demyelinating polyneuropathy [23].

Since the start of the coronavirus disease 2019 (COVID-19) pandemic, neurological manifestations related to severe acute respiratory syndrome coronavirus 2 (SARS-CoV-2) infection have included rare case reports and case series of GBS with bilateral facial nerve palsy. A systematic literature review focusing on bilateral facial diplegia as a neurological sequela of COVID-19 infection found 15 patients with facial diplegia associated with SARS-CoV-2 infection, and a majority of cases had favorable outcomes with good recovery. Hence, coronaviruses can be recognized as another potential trigger for GBS [24].

In the United States, 132 GBS cases were reported among adenovirus (ad26.COV2.S) vector vaccine recipients after administering 13.2 million doses (Janssen/Johnson & Johnson). The rate was 9.8 cases per million doses, which is four times the background rate. The median time to onset was 13 days following vaccination, and 35% had a life-threatening case. In an earlier report of 100 cases, a quarter of the patients reported facial diplegia [25].

The limitations of this study include its retrospective design and small sample size as the facial diplegia variant of GBS is an extremely rare condition. This study can help neurologists in evaluating patients with bilateral facial muscle weakness and localizing lesions, either in the peripheral (facial and trigeminal nerves) or central (pons and medulla) nervous system. In patients with facial diplegia, electrodiagnostic studies such as direct facial nerve conduction stimulation, blink reflex, and needle EMG of facial muscles along with nerve conduction studies of all four limbs are important diagnostic investigations for the underlying pathologic process of the nerves (axonal, demyelinating, or mixed) and for localizing lesions.

## Conclusions

Facial diplegia, a variant of GBS, is an extremely rare condition. Bilateral facial nerve palsy may occur in central or peripheral lesions with diverse etiologies. The most common cause of facial diplegia in this study was GBS. Electrodiagnostic studies including direct facial nerve conduction, blink reflex, and concentric needle EMG of facial muscles are important prognostic markers for patients with facial diplegia as well as help in lesion localization to peripheral (trigeminal and facial nerves) or central (pons and medulla) nervous system.
